# Resposta às contribuições de Francisco Paumgartten ao texto “Maria José Deane por ela mesma e por nós”, publicado no volume 32^*^

**DOI:** 10.1590/S0104-59702026000100030

**Published:** 2026-08-10

**Authors:** Laurinda Rosa Maciel, Renata Silva Borges

**Affiliations:** i Tecnologista em saúde pública, Departamento de Arquivo e Documentação/Casa de Oswaldo Cruz/Fiocruz. Rio de Janeiro – RJ – Brasil. laurinda.maciel@fiocruz.br; ii Tecnologista em saúde pública, Departamento de Arquivo e Documentação/Casa de Oswaldo Cruz/Fiocruz. Rio de Janeiro – RJ – Brasil. renata.borges@fiocruz.br

Agradecemos as importantes contribuições do leitor ao texto “Maria José Deane por ela mesma e por nós” (Maciel, Borges, 2025), publicadas na seção Carta ao Editor da revista *História, Ciência e Saúde – Manguinhos* (Paumgartten, 2026). Compreendemos que as imprecisões apontadas procedem e que as fontes primárias consultadas pelo leitor são oportunas e decorrem do caminho percorrido a partir de sua leitura do artigo cotejada com outras fontes, possivelmente elencando escolhas distintas das que percorremos até chegarmos ao texto originalmente publicado.

Com isso, demarcamos o nosso ponto de vista a partir dos registros que selecionamos como fontes e dos contextos nos quais nos inserimos como leitoras, profissionais e pesquisadoras. Nesse sentido, qualquer texto que vier a refutar, corrigir ou ampliar as informações sobre a história de Maria Deane e sua trajetória será bem-vindo e se apresentará como mais uma contribuição, o que é muito benéfico para o campo da história.

As informações que divulgamos no texto são baseadas em fontes citadas em seu escopo e devidamente referenciadas, com destaque para a entrevista, única fonte primária escolhida. A produção do texto ocorreu baseada em escolhas de fontes validadas cientificamente e em metodologia que possibilitasse sua apresentação final em formato mais adequado à sua publicação na seção Fontes da revista. A submissão e a avaliação do escrito incluíram criteriosa avaliação pelos pares e tratamento final condizente com a qualidade preconizada pelo periódico.

Em razão dos distintos caminhos seguidos por autores e leitores, concluímos que as imprecisões são possíveis e previsíveis, sobretudo quando advindas de diferentes perspectivas e olhares que contêm práticas particulares. Esse diálogo entre leitor e autor é sempre oportuno e certamente contribuirá para a produção de novos conhecimentos sobre o tema do artigo. Assim, reiteramos nosso agradecimento ao leitor e esperamos que as imprecisões apontadas sejam acolhidas em novos textos sobre Maria Deane.
